# Attenuated Salmonella carrying plasmid co-expressing HPV16 L1 and siRNA-E6 for cervical cancer therapy

**DOI:** 10.1038/s41598-021-99425-3

**Published:** 2021-10-11

**Authors:** Junyu Chen, Shuhua Zhao, Wenxi Tan, Taiwei Wang, Shan Wu, Changshuai Wang, Yu Jiang, Tuo Zhou, Zhuo Zhang, Lijing Zhao

**Affiliations:** 1 Department of Rehabilitation, School of Nursing, Jilin University, 965 Xinjiang Street, Changchun, China; 2 Department of Gynecology, Second Hospital, Jilin University, Changchun, China; 3 Key Laboratory of Reproductive Genetics (Ministry of Education) and Department of Reproductive Endocrinology, Women’s Hospital, Zhejiang University, School of Medicine, Zhejiang, China

**Keywords:** Cancer, Genetics, Immunology

## Abstract

Human papillomavirus (HPV) infection is the major etiological factor for cervical cancer. HPV prophylactic vaccines based on L1 virus-like particles have been considered as an effective prevention method. However, existing recombination vaccines are too expensive for developing countries. DNA vaccines might be a lower-cost and effective alternative. In this study, a plasmid (pcDNA3.1-HPV16-L1) and a co-expressing plasmid (pcDNA3.1-HPV16-L1-siE6) carried by attenuated Salmonella were constructed and their prevention and treatment effect on cervical cancer were observed, respectively. The results showed that pcDNA3.1-HPV16-L1 carried by attenuated Salmonella could induce the production of HPV16-L1 antibodies, IL-2 and INF-γ in mice serum, which presented its prevention effect on HPV. Subsequently, E6 and E7 gene silencing by pCG-siE6 inhibited the growth of cervical cancer both in vitro and in vivo. Furthermore, L1 up-regulation and E6/E7 down-regulation caused by co-expressing plasmid (pcDNA3.1-HPV16-L1-siE6) contributed to a significant anti-tumor effect on the mice. This study suggests that pcDNA3.1-HPV16-L1-siE6 carried by attenuated Salmonella has a synergistic effect of immune regulation and RNA interference in cervical cancer treatment.

## Introduction

Cervical cancer is the predominant cancer in developing countries among women^1^. 86% of cervical cancer cases and 88% of mortalities caused by cervical cancer occur in developing countries^2^. Despite of the widespread screening and vaccine implementation, there are still approximately 54,000 and 11,000 cases each year in Europe and USA, respectively^2,3^. The standard treatment of advanced cervical cancer is radical surgery or chemoradiation, but the quality of life in multiple cases is still poor^4^. Hence, the exploration of specific strategy for the prevention and early treatment of cervical cancer is urgently needed.

Cervical cancer is widely considered as the outcome of high-risk human papillomavirus (HR HPV) infections^5^. HPV16 is identified as the most prevalent type and detected in more than 50% of cervical cancer cases^6^. The genome of HPV consists of a circular double-stranded DNA including non-coding control regions (NCR), early region (E) coding E1, E2, E4, E5, E6 and E7 genes, and late region (L) coding L1 and L2 genes^7,8^. L1 and L2 proteins are structure proteins for HPV capsid. Under specific conditions, L1 protein can self-assemble to virus-like particles (VLPs) with a strong immunogenicities without infectious and carcinogenic abilities^5^. Currently, three HPV prophylactic vaccines based on L1 VLPs have been widely used in developed countries and showed a desired reduction of 38% in high grade dysplasia^9^. However, the developing countries with high incident rates are not able to implement these vaccines due to the high cost and requirement of multiple injections^10^. Thus, control measures against HPV around the world still require lower-cost prophylactic vaccines and therapeutic alternatives^11^.

E6 and E7 proteins play a crucial role in cervical carcinogenesis through disrupting important cell pathways such as ubiquitin-mediated degradation of tumor suppressor protein P53^4,12^. Many studies showed that silencing E6 and/or E7 gene by small interference RNA (siRNA) can significantly inhibit the development of cervical cancer in vitro or in vivo^13–15^. However, the absence of vectors that can stably transmit siRNA into target cells limits their clinical applications^16^. Attenuated Salmonella can easily accumulate and proliferate in the tumor microenvironment^17^. Moreover, alive attenuated Salmonella can induce mucosal immune response^10^. Therefore, attenuated Salmonella is considered as a promising vaccine vector for cervical cancer prevention and therapy. A previous study testified that a HPV16-L1 expressing plasmid carried by attenuated Salmonella (Ty21a) successfully induced HPV16 neutralizing antibodies in serum and genital secretions in mice model^10^.

In this study, pcDNA3.1-HPV16-L1 carried by attenuated Salmonella (Ty21a or PhoP/PhoQ) was initially constructed and significantly induced the production of HPV16-L1 antibody in serum and genital secretions of mice through intranasal dripping. After the anti-tumor effect of pGC-siE6 had been verified, pcDNA3.1-HPV16-L1-siE6 plasmid carried by attenuated Salmonella (PhoP/PhoQ) was further conducted, and its effect of therapy on cervical cancer was observed.

## Results

### Expression of HPV16-L1 in BHK cells

**Figure 1:**
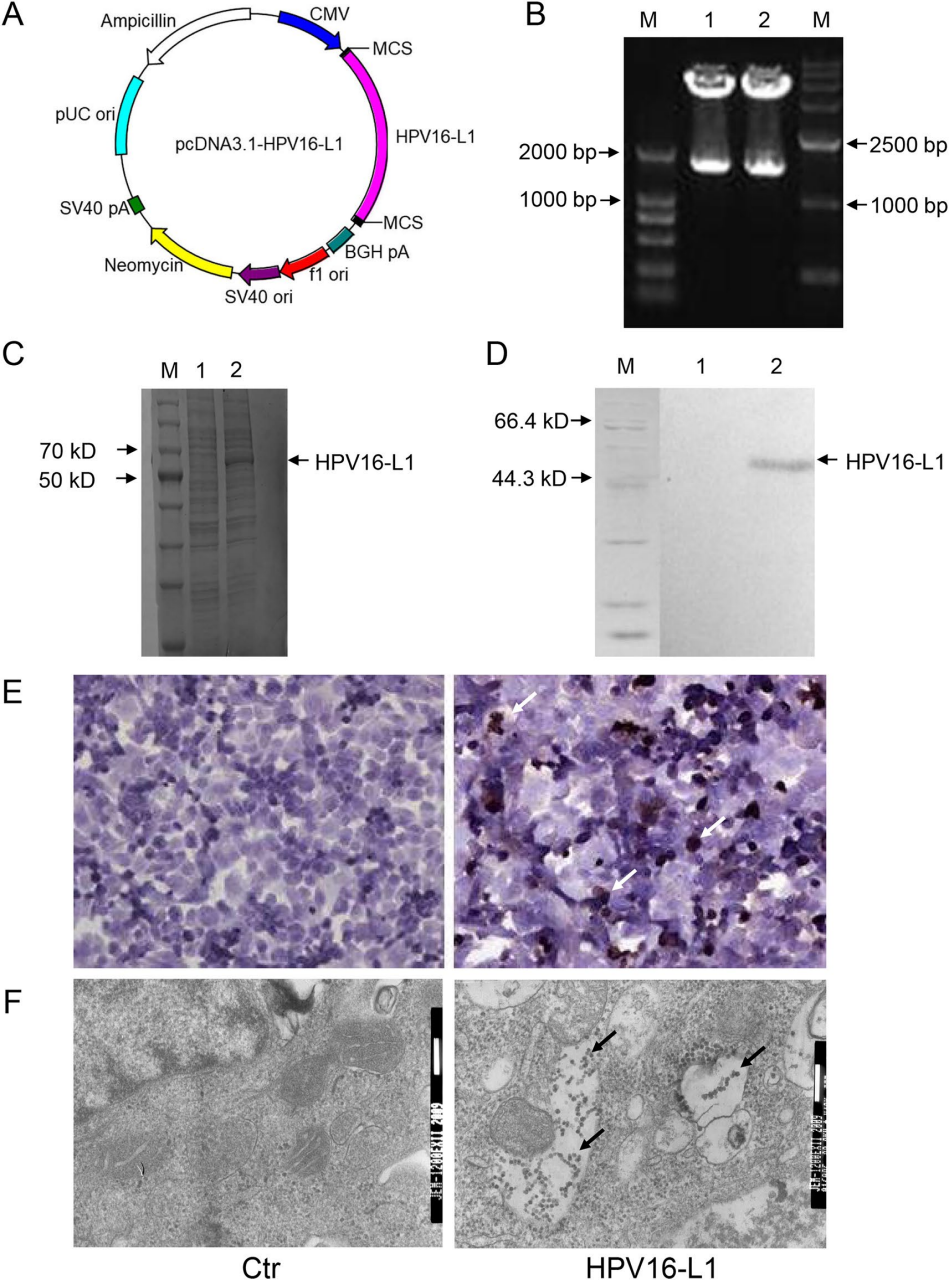
The pcDNA3.1-HPV16-L1 plasmid was successfully constructed and HPV16-L1 was identified to express in BHK cells. (**A**) The map of pcDNA3.1-HPV16-L1. (**B**) Identification of pcDNA3.1-HPV16-L1 by restriction enzyme digestion. (M1: DL2 000; lane 12: plasmid digested by HindIII and KpnI; M2: DL15000). (**C**) SDS PAGE analysis of HPV16-L1 protein expressed in BHK cells (M: protein marker, lane 1: control, lane 2: expressed protein). (**D**) Western blotting analysis of HPV16-L1 protein expressed in BHK cells (M: protein marker; lane 1: control; lane 2: expressed protein). (**E**) Immunocytochemistry analysis of HPV16-L1 protein expressed in BHK(× 400). (**F**) Electron microscropy detection of HPV16-L1 expressed in BHK cells.

pcDNA3.1-HPV16-L1 was constructed (Fig. 1A) and confirmed by double enzyme digestion. As shown in Fig. 1B, the obvious stripes representing HPV16-L1 gene were presented in 1500 bp. The results of SDS PAGE, Western blot, and Immunocytochemistry showed the positive expression of HPV16-L1 in transfection group and the negative expression in control group in BHK cells (Fig. 1C–E). Furthermore, L1 VLP was observed in the transfection group under electron microscope (Fig. 1F). These results indicated that HPV16-L1 protein was successfully expressed and formed into self-assembly VLPs in BHK cells.

### pcDNA3.1-HPV16-L1 carried by attenuated Salmonella activated the immune responses of mice

**Figure 2:**
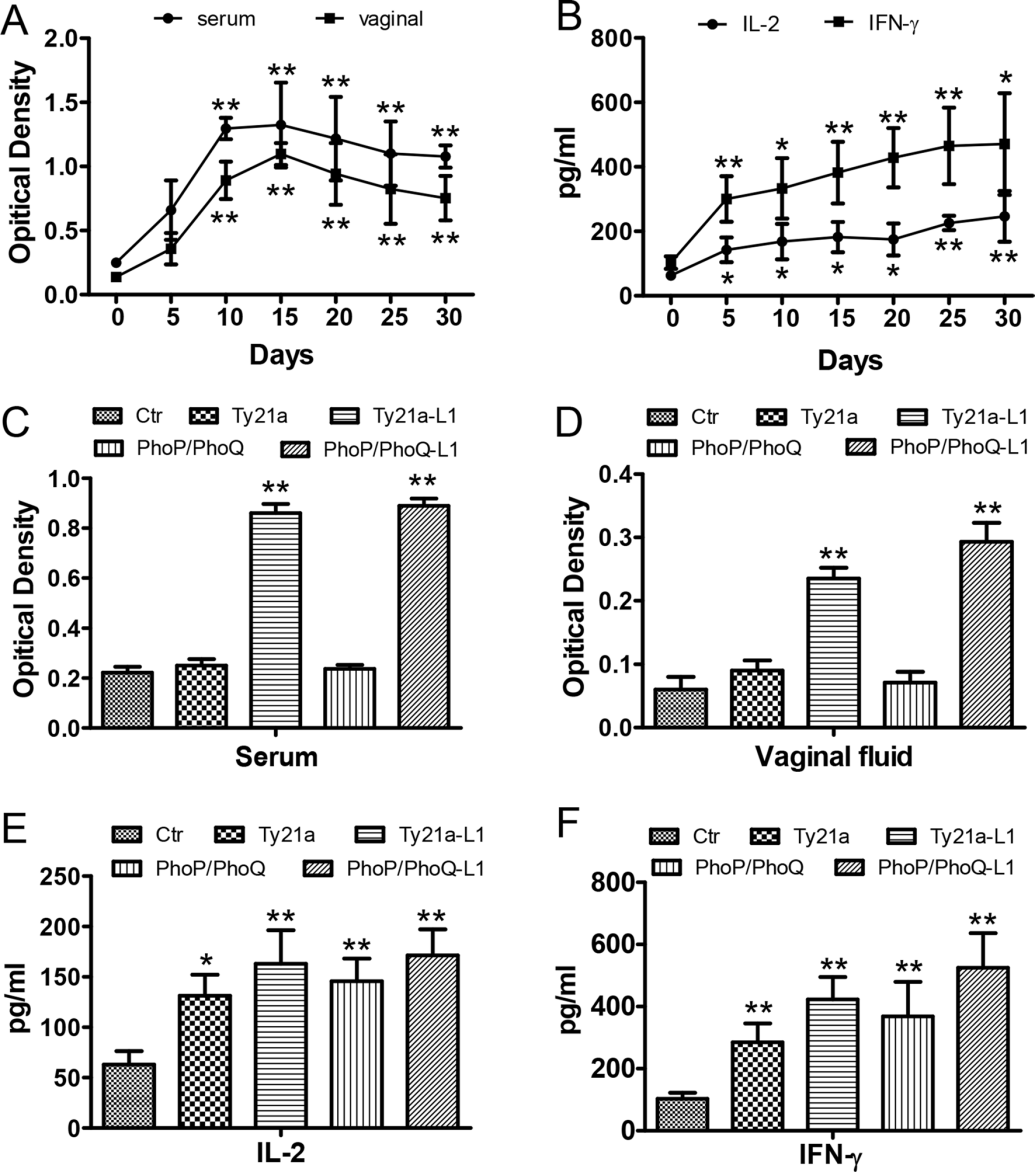
After immune with attenuated Salmonella carrying pcDNA3.1-HPV16-L1, antibodies were produced and cytokines were increased in mice. (**A**) ELISA detection of anti-HPV16-L1 antibody after vaccinated 1 time. *P < 0.05, **P < 0.01, vs the 0d group. (**B**) ELISA detection of IL-2 and IFN-γ after vaccinated 3 times. *P < 0.05, **P < 0.01, vs the 0d group. (**C**,**D**) ELISA detection of anti-HPV16-L1 antibody after vaccinated 3 times. **P < 0.01, vs the control group. (**E**,**F**) ELISA detection of IL-2 and IFN-γ after vaccinated 3 times. *P < 0.05, **P < 0.01, vs the control group.

After being treated by attenuated Salmonella carrying pcDNA3.1-HPV16-L1 plasmid, the serum and genital secretions of mice were collected for ELISA assay. The results indicated that anti-HPV16-L1 in serum and genital secretions started to increase on day 10 after being vaccinated once, and maintained at a high level for at least 30 days (Fig. 2A). Compared with 0 day group, IL-2 and IFN-γ were increased after the treatment (Fig. 2B).

Moreover, after being vaccinated for three times, anti-HPV16-L1 antibody in serum and genital secretions were significantly increased (P < 0.05) both in phoP/phoQ (pcDNA3.1-HPV16-L1) and Ty21a (pcDNA3.1-HPV16-L1) groups as compared to control groups, respectively (Fig. 2C,D). Interestingly, IL-2 and IFN-γ in mice serum were all significantly increased in the groups vaccinated with phoP/phoQ (pcDNA3.1-HPV16-L1) or Ty21a (pcDNA3.1-HPV16-L1) for three times (Fig. 2E,F). These results implied that pcDNA3.1-HPV16-L1 carried by attenuated Salmonella activated the immune system of mice.

### pGC-siRNA down-regulated E6 and E7 expression in Siha cells

**Figure 3:**
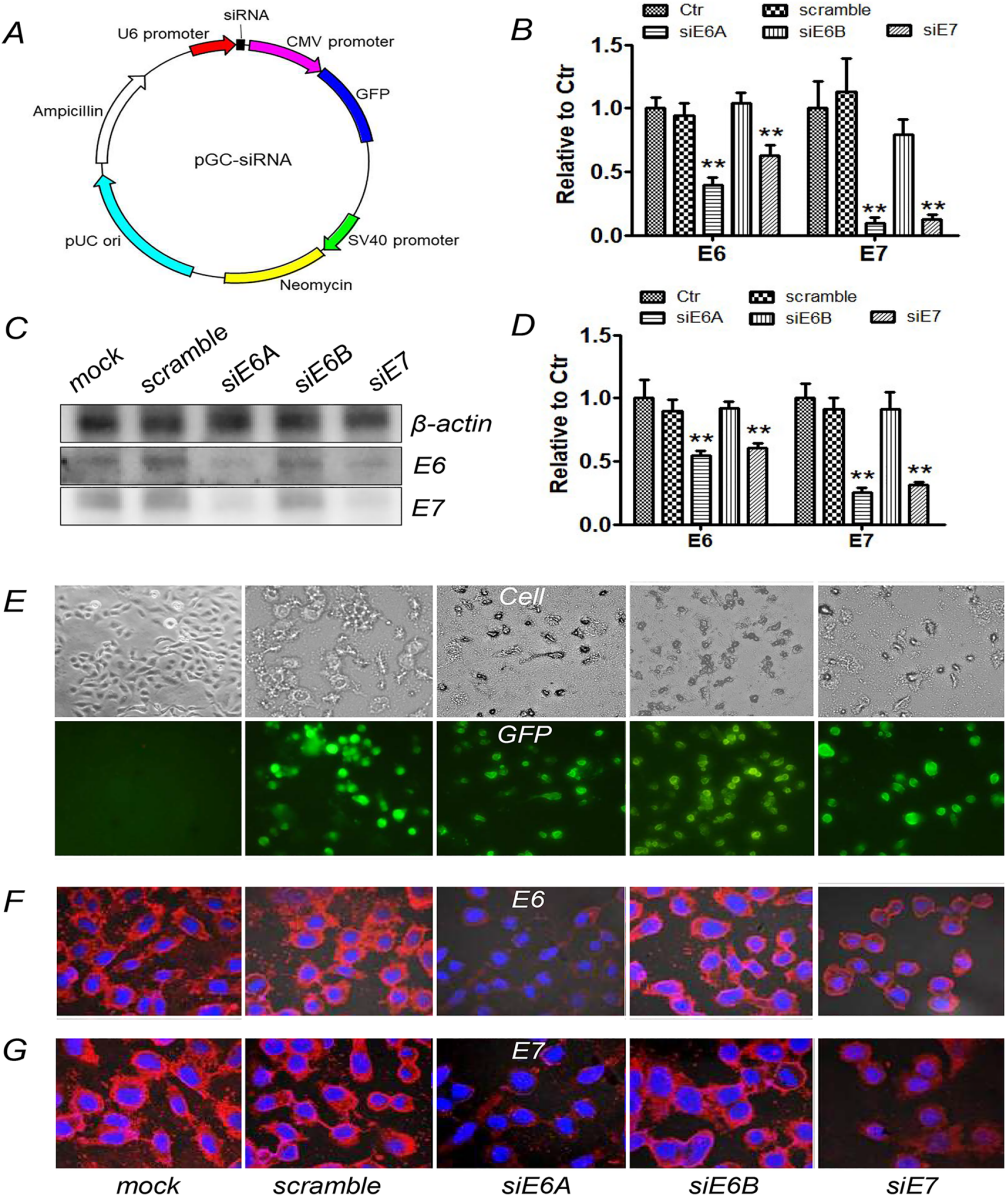
After transfection with pGC-siE6A or pGC-siE7 plasmid, the expression of HPV-E6/E7 protein was decreased in Siha cells. (**A**) The map of pGC-siRNA. (**B**) Semi-quantitative RT-PCR analysis of HPV16-E6/E7 mRNA in Siha cells transfected with pGC-siRNA plasmid. (**C**,**D**) Western blotting analysis of HPV16-E6 and HPV16-E7 protein in Siha cells transfected with pGC-siRNA plasmid. (**E**) Transfection efficiency determined by fluorescence microscope. (**F**,**G**) Immunofluorescence analysis for HPV16-E6/E7 protein in Siha cells transfected with pGC-siRNA plasmids (× 600).

The results of fluorescence microscope showed that pGC-siRNAs (Fig. 3A) were transfected into Siha cells with high transfection efficiency (Fig. 3E) and down-regulate the red fluorescence intensity representing E6 or E7 expression in pGC-siE6A and pGC-siE7 groups compared with mock and pGC-scramble groups (Fig. 3F,G). The results of RT-PCR and Western blot were in accordance with the results of Immunofluorescence (Fig. 3B–D). These results indicated that pGC-siE6A and pGC-siE7 could down-regulate the expression of E6 and E7 in Siha cells.

### pGC-siRNA inhibited Siha cells through inducing P53-mediated apoptosis pathway in vitro

**Figure 4:**
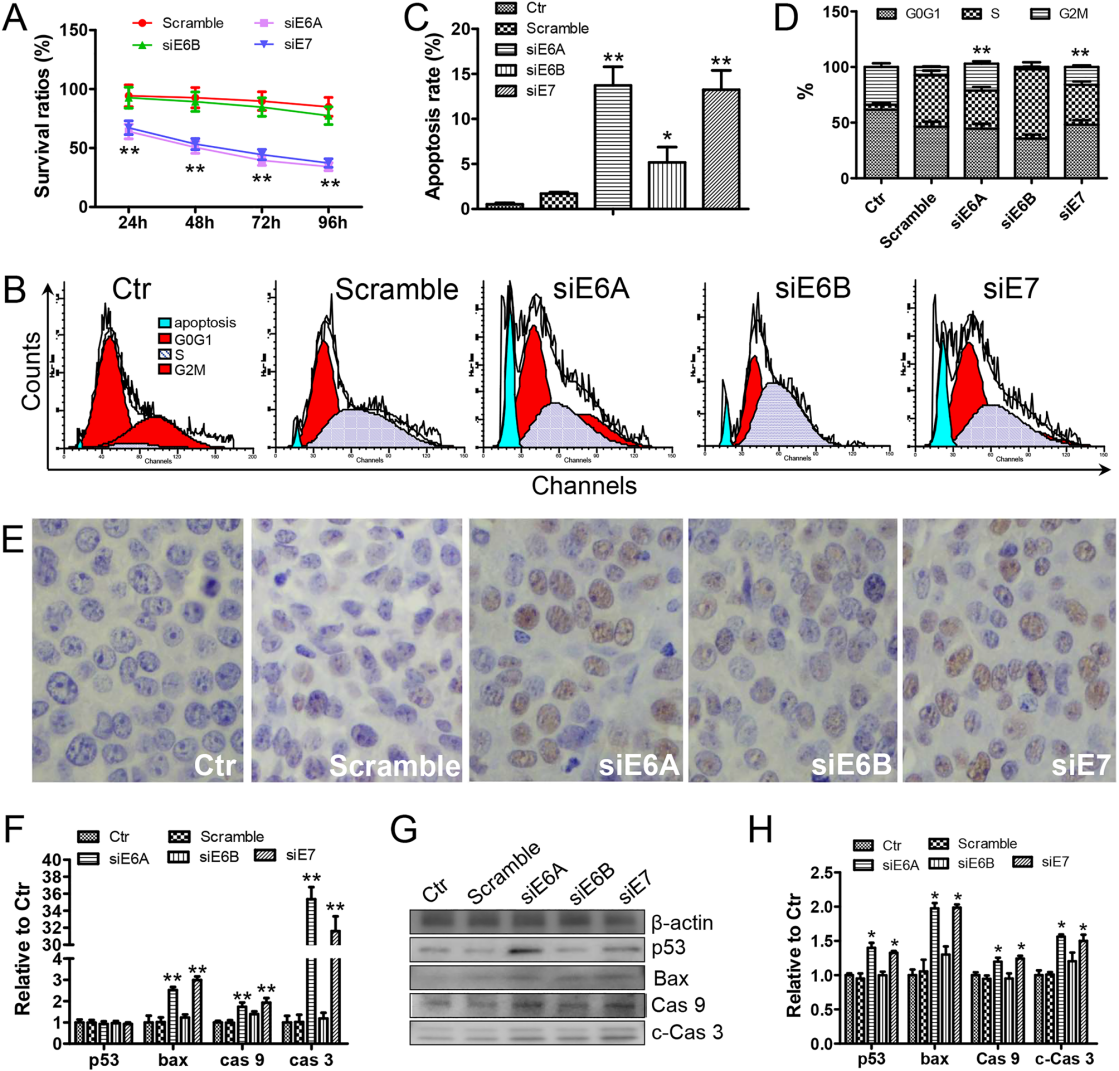
The pCG-siE6A and pCG-siE7 can promote the apoptosis of Siha cells. (**A**) Survival ratios for Siha cells transfected with pGC-siRNA plasmids (n = 3). **P < 0.01, vs the control group. (**B**,**C**) FCAS analysis for apoptosis rates of Siha cells transfected with pGC-siRNA plasmids. *P < 0.05, **P < 0.01, vs the two control groups. (**D**) FCAS analysis for cell cycle of Siha transfected with pGC-siRNA plasmids. **P < 0.01, vs the Scramble group. (**E**) TUNEL analysis for Siha cells transfected with pGC-siRNA plasmids (× 400). (**F**) Semi-quantitative RT-PCR analysis of mRNA in Siha cells transfected with pGC-siRNA plasmids, **P < 0.01, vs the two control groups. (**G**,**H**) Western blotting analysis of related protein in Siha cells transfected with pGC-siRNA plasmids, *P < 0.05, vs the two control groups.

MTT, TUNEL, and flow cytometry were used to evaluate the anti-tumor effect of the pGC-siRNA on Siha cells. As shown in Fig. 4A, the survival rations of the cells treated with pGC-siE6A or pGC-siE7 were significantly lower than the cells treated with pGC-scramble. Furthermore, the results of TUNEL assay and flow cytometry revealed that the apoptosis rates in pGC-siE6A or pGC-siE7 group were significantly higher than those in control or pGC-scramble group (Fig. 4B,C,E). After treatment with pGC-siE6A and pGC-siE7 plasmids, the cell cycle of Siha was blocked in G2 phase (Fig. 4B,D).

The expressions of P53, Bax, and Caspase family were examined by RT-PCR and Western blot. As shown in Fig. 4F, pGC-siE6A or pGC-siE7 significantly (P < 0.01 = increased the transcription of bax, caspase9, and caspase3. In accordance with these results, the expression of Bax, Caspase9, and cleaved-Caspase3 (c-Caspase3) proteins was significantly (P < 0.05) up-regulated in the cells transfected with pGC-siE6A or pGC-siE7 (Fig. 4G,H). Interestingly, pGC-siE6A or pGC-siE7 still up-regulated the expression of P53 protein in Siha cells, suggesting that P53 might be post-transcriptionally regulated by E6/E7 (Fig. 4G,H). These results suggested that pGC-siRNA could inhibit Siha cells through inducing P53-mediated apoptosis pathway.

### pGC-siE6A inhibited the growth of xenografts in vivo

**Figure 5:**
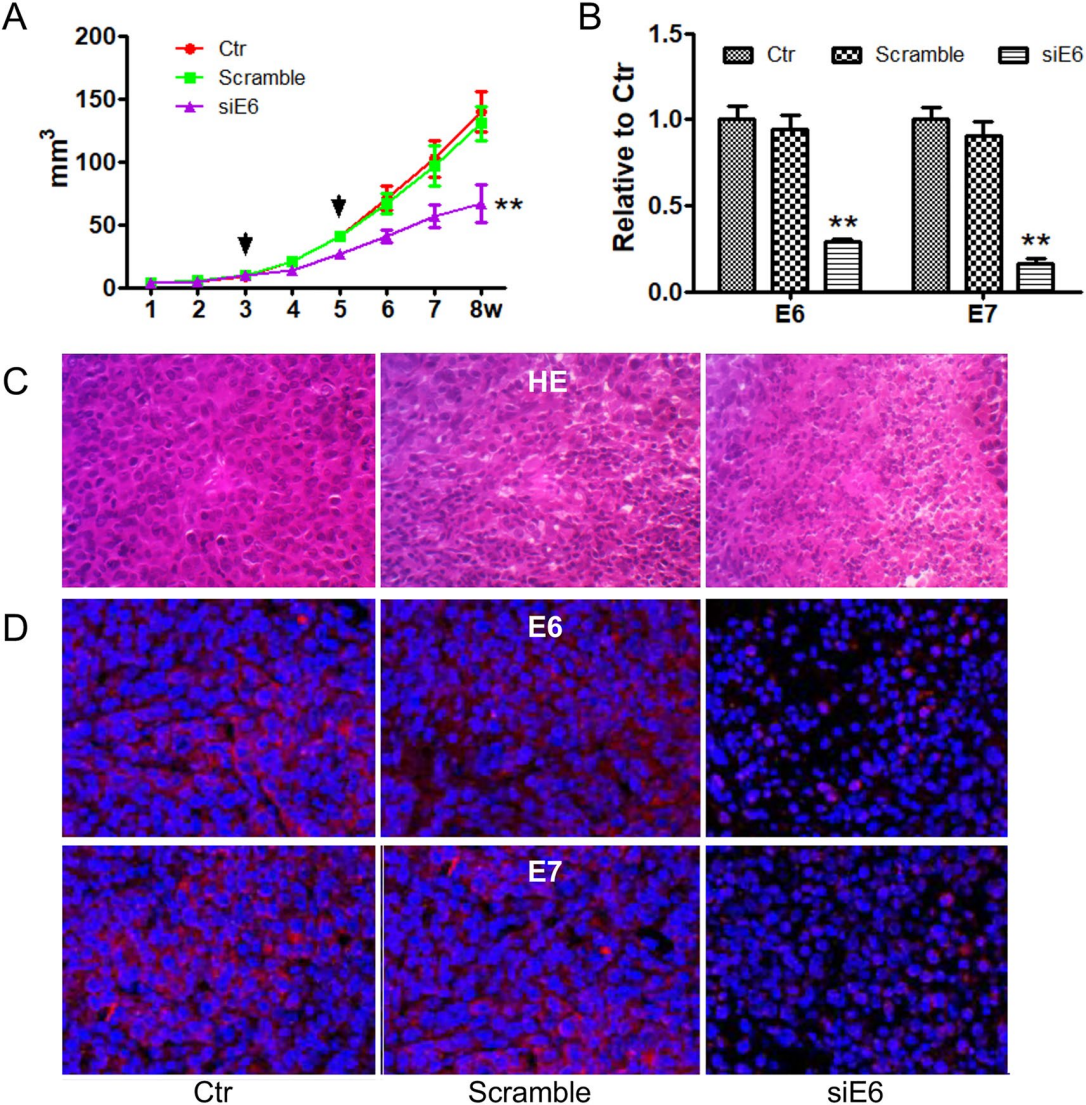
pCG-siE6 can inhibit the growth of the xenograft tumors in vivo. (**A**) Growth of Siha xenografts treated with pGC-siE6 (n = 5). Each treatment with the plasmid is shown an arrow. **P < 0.01, vs the two control groups. (**B**) Semi-quantitative RT-PCR analysis of HPV16-E6/E7 mRNA in Siha xenografts treated with pGC-siE6 plasmids. **P < 0.01, vs the two control groups. (**C**) HE assay for Siha xenografts treated with pGC-siE6 plasmids (× 400). (**D**) Fluorescence immunohistochemical analysis of HPV16-E6/E7 protein expression in Siha xenografts treated with plasmids (× 600).

To evaluate the effects of pGC-siE6A on tumor growth in vivo, an orthotopically xenograft model was monitored. As shown in Fig. 5A, the volume of tumors in pGC-siE6A group was significantly (P < 0.05) decreased compared with control and pGC-Scramble groups.

The results of RT-PCR showed that the transcription of E6 and E7 in pGC-siE6A group was down-regulated (P < 0.05) compared to control and pGC-Scramble groups (Fig. 5B). H&E staining showed more cell debris and death cells featured with nuclear consolidation, fragmentation, and disintegration in pGC-siE6 group (Fig. 5C). Furthermore, red fluorescence intensity representing E6 or E7 protein was significantly weakened in pGC-siE6 group (Fig. 5D). These results suggested that silencing E6 and E7 gene could inhibit the xenograft tumor growth.

### pcDNA3.1-HPV16-L1-siE6 carried by attenuated Salmonella inhibited the growth of xenografts

**Figure 6:**
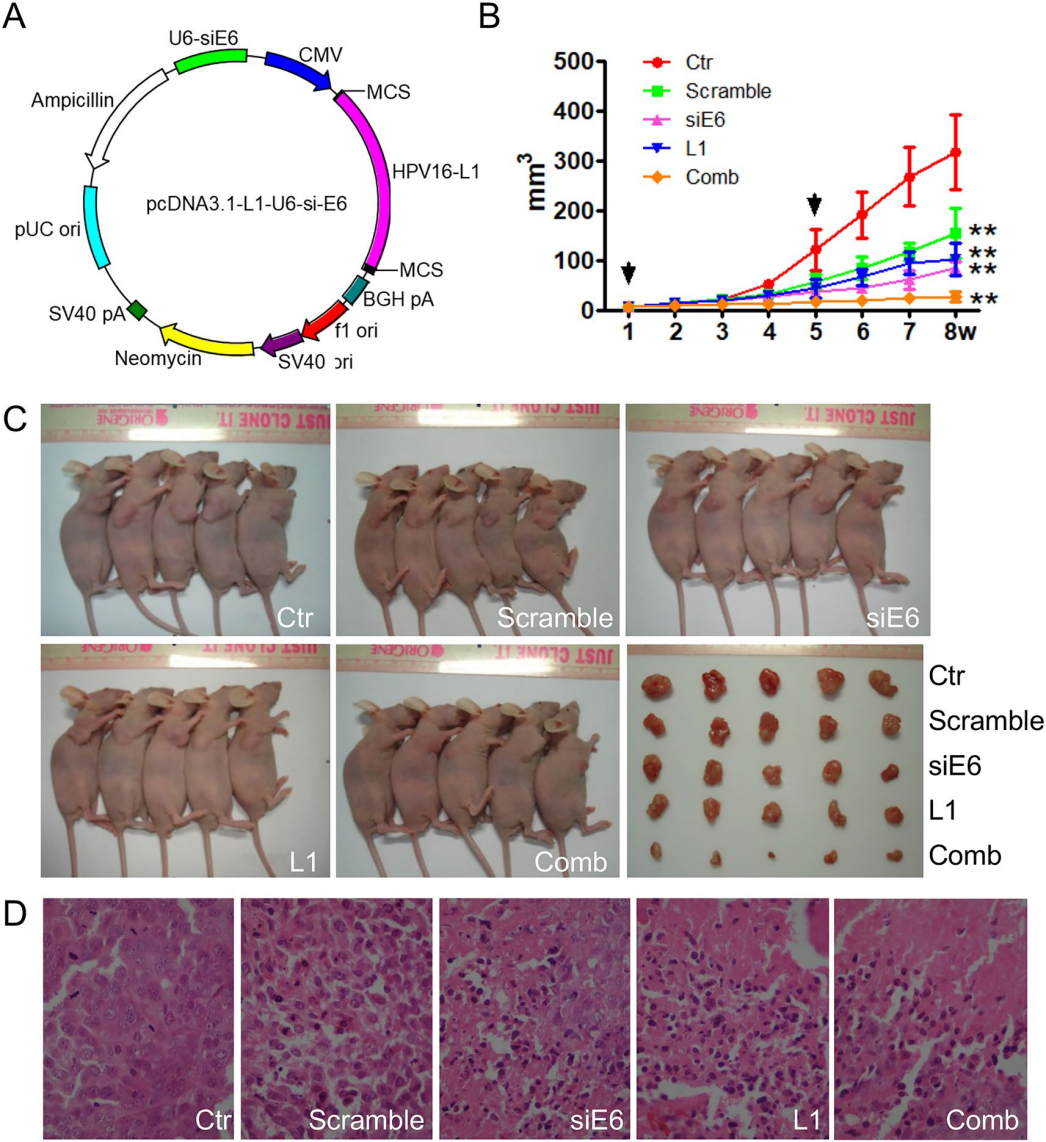
The phoP/phoQ(pcDNA3.1- L1-siE6) can inhibit the growth of the xenograft tumors in vivo. (**A**) The map of pcDNA3.1- L1-siE6. (**B**,**C**) Growth of Siha xenografts treated with attenuated Salmonella phoP/phoQ. Each treatment with the plasmid is shown an arrow. **P < 0.01, vs the two control groups. (**D**) H&E analysis of Siha xenografts treated with phoP/phoQ (× 400).

To investigate whether the combination of HPV16-L1 expression and E6/E7 down-regulation affected cervical xenograft proliferation on mice, initially performed pcDNA3.1-HPV16-L1-siE6 (Fig. 6A) carried by attenuated Salmonella phoP/phoQ was used in this study. As demonstrated in Fig. 6B,C, the tumor sizes in combination group were significantly decreased compared with HPV16-L1 or siRNA-E6 group. In addition, the tumor growth was also significantly inhibited in HPV16-L1 group compared with control and siRNA-Scramble groups, respectively (Fig. 6B,C). Histological examination with H&E staining presented necrotic cells along with tissue disorganization in the siRNA-E6, HPV16-L1 and combination treatment groups (Fig. 6D).

**Figure 7:**
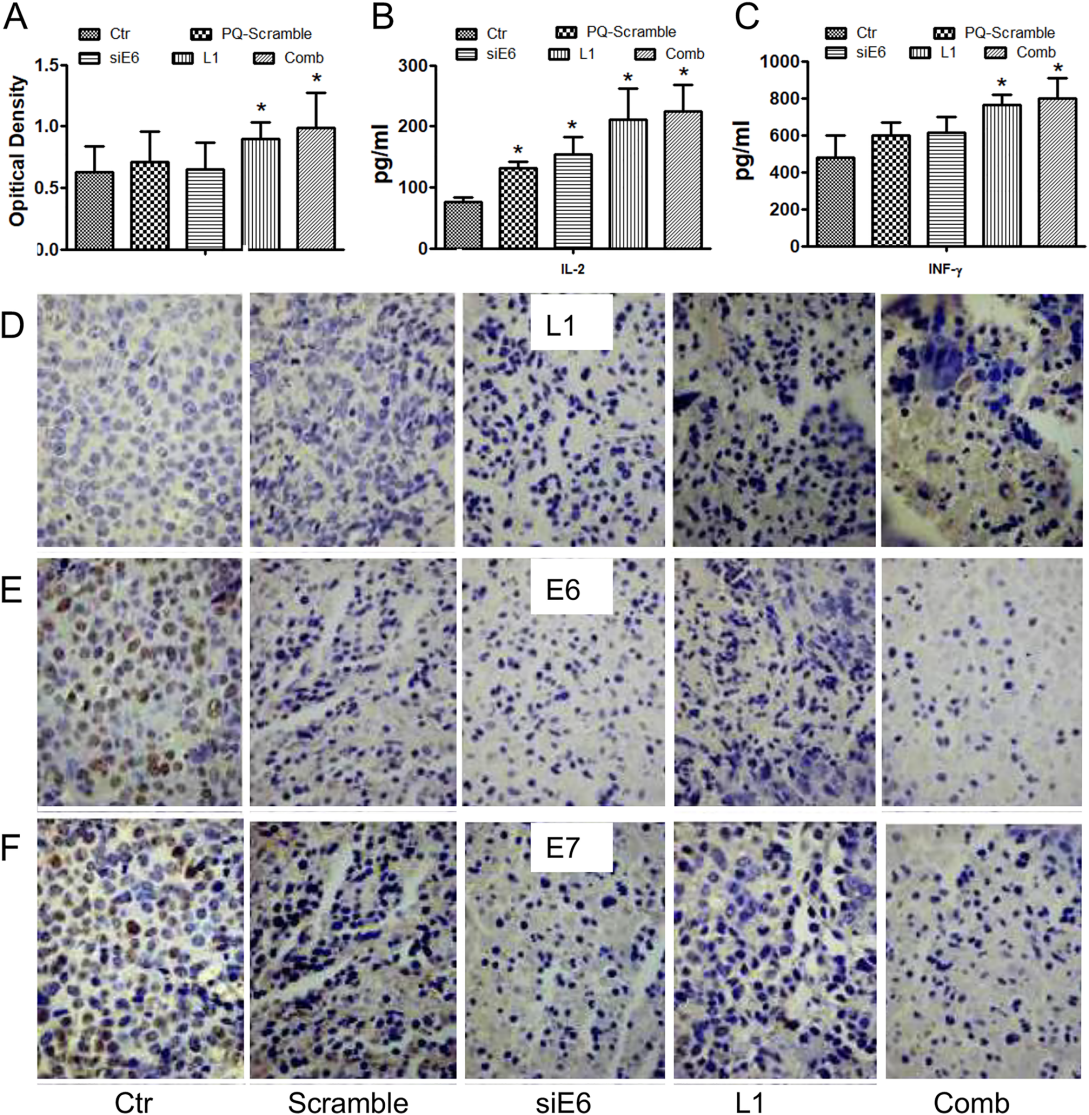
The mechanism of phoP/phoQ(pcDNA3.1-L1-siE6) inhibiting the growth of xenograft tumors in vivo. (**A**) ELISA analysis of anti-HPV16-L1 antibody in serum after treated with phoP/phoQ. *P < 0.05, vs the control group. (**B**,**C**) ELISA anal-ysis of IL-2 and IFN-γ in serum after treated with phoP/phoQ. *P < 0.05, vs the control group. (**D**–**F**) Immunohistochemical analysis of HPV-L1/E6/E7 protein expression in Siha xenografts treated with phoP/phoQ (× 400).

Moreover, when compared to the control group, anti-HPV16-L1, IL-2 and IFN-γ in serum and HPV16-L1 protein in tumor tissues was robustly increased in the HPV16-L1 and combination groups (Fig. 7A–D). Both the expression of E6 and E7 in the combination group and siRNA-E6 group were significantly down-regulated (P < 0.05) compared with that in the other three groups (Fig. 7E,F).

## Discussion

Globally, cervical cancer is the third frequent cause of cancer-related death among women^18^, and is considered as a primary disease that shortens women’s lifespan, especially in developing countries^19^. The standard treatment of cervical cancer is mainly based on surgery and radiotherapy supplemented by chemotherapy, which is predisposed to severe side effects. It is urgently to develop more novel methods to prevent and treat cervical cancer.

More than 100 types of HPV have been identified based on its various L1 gene sequence^20^. HPV 16 has been detected in 57.4% of cervical cancer in Australia^21^. Unlike the current HPV vaccine, the productive processes of DNA vaccine could be easier and cheaper. Importantly, the potential immunogenicity and the ability synergically combining with gene silencing tools are features achieved by DNA vaccine, which might contribute to cervical cancer therapy. Many HPV immunotherapies successfully induce an immune response against epitopes of HPV16 E6/E7 antigens and inhibit tumor growth in experimental mouse models^22^. However, since high-risk HPV E6/E7 proteins may lead to an aggressive proliferation in normal cells, inhibiting the expression of E6/E7 through RNA interference has been confirmed to achieve considerable treatment effects on cervical cancer^23^. Unfortunately, the absence of vector that stably transfers the genes into targeted cells limits the application of this technology^17^.

Attenuated Salmonella, the first recombinant bacteria used to delivery antigen^24^, can express foreign antigens and induce systemic immune responses after being vaccinated through nasal mucosa^25^. In cancer treatment, attenuated Salmonella have an outstanding safety trait, by predisposing to targeted tumors and growing under hypoxic tumor microenvironment^26^. The biological advantages make it to be a promising vaccine vector for the cervical cancer prevention and therapy.

In this study, a low-cost DNA vaccine was designed. The results showed that pcDNA3.1-HPV16-L1 transfection successfully induced HPV16-L1 expression and VLP assemble in BHK cells. Although the effects of many HPV16-L1-based DNA vaccines have been testified, the degree of immunity depends on the choice of vector, immune pathway, and molecular adjuvants.

As an intracellular parasitic bacterium, attenuated Salmonella can express natural mucosal immune adjuvant and exogenous antigen that effectively elicits immune cells activation, cytokine secretion, cytotoxic lymphocyte (CTL) production and specific antibody formation^10^. IL-2 is a key regulator in immune system, which can induce the production of interferon-γ (IFN-γ) contributing to the virus and tumor therapy through cellular immunity^27^. Furthermore, the expression of IL-2 and IFN-γ up-regulated by antigen presenting cell (APC) could enhance the immune response induced by DNA vaccine^27–30^. In this study, pcDNA3.1-HPV16-L1 carried by attenuated Salmonella presented well features as a vector with increased anti-HPV16-L1 antibody, IL-2, and IFN-γ. Hence, we supposed that after this DNA vaccine was engulfed by APC, HPV16-L1 protein was expressed and stimulated B lymphocytes to mature and produce anti-HPV16-L1 antibodies. Simultaneously, the activated APC also promoted the release of IL-2 and IFN-γ, which might play an important role against viruses and tumors. The data above implied that pcDNA3.1-HPV16-L1 carried by attenuated Salmonella might be an effective DNA vaccine for HPV prevention. Interestingly, we also found this pcDNA3.1-HPV16-L1 carried by attenuated Salmonella could inhibit cervical tumor growth in vivo, which implied the DNA vaccine might be a potential treatment strategy. However, this treatment effect has rarely been observed in the other VLP vaccines. We just cautiously supposed the features that attenuated Salmonella carrying pcDNA3.1-HPV16-L1 was easier to accumulate and express HPV16-L1 protein in tumor tissues compared with other VLP vaccines contributed to this anti-tumor effect.

It is well established that the continuous expression of E6 and E7 proteins in cervical cells triggers and promotes the carcinogenesis of cervix^4,12^. In non-infected cervical cells, cellular ubiquitin ligase, E6AP (E6 associated protein) does not affect P53 who triggers cell cycle arrest to induce apoptosis^31,32^. However, with continuous HPV16 infection, E6 exerts its ubiquitin-mediated p53-degration effects by interacting with E6AP in cervical cells^33^. Bax protein and Caspase family proteins are crucial mediators and symbols of cell apoptosis regulated by P53^17,34^. c-Caspase9 and c-Caspase3 can trigger the caspase cascade and process of apoptosis^35^. The results in this study showed that the expression of E6 and E7 was down-regulated by pGC-siE6 in vitro or in vivo, resulting in a marked anti-tumor effect. Additionally, E6 gene silencing inhibited the development of cervical cancer through activating p53-mdiated apoptosis pathway including the up-regulation of Bax, Caspase9, and c-Caspase3. These results identified that pGC-siE6, one part of the recombination vaccine, restrained the degradation of P53 protein and then initiated the apoptosis pathway, which finally inhibited the development of cervical cancer.

In order to expand the range of application and enhance the anti-tumor effect of the vaccine, a co-expression plasmid vaccine, pcDNA3.1-HPV16-L1-siE6 carried by attenuated Salmonella was constructed. After being vaccinated with the co-expression plasmid vaccine through nasal dripping, HPV16-L1 gene expression was induced whereas E6/E7 gene expression was silenced in the tumor tissues. Due to the expression of HPV16-L1 induced by the co-expression plasmid vaccine, B lymphocytes produced anti-HPV16-L1 antibodies and precisely target the cervical tumor in mice. Previous studies had shown that secretion of pro-inflammatory cytokines, including IL-2 and IFN-γ, is observed in macrophage activation and then induced anti-tumor activity on human cervical cancer cells^36^. Therefore, we also suggested that the APC cells of tumor-bearing mice were activated by engulfing the co-expression plasmid vaccine and then up-regulated the levels of IL-2 and IFN-γ both in serum and genital secretions. Collectively, the vaccine stimulated both of the humoral immunity and cellular immunity through inducing HPV16-L1 expression in the murine tumor tissues and increasing the levels of IL-2 and IFN-γ in serum and genital secretions. It is therefore possible to inhibit the tumor size through activating immune pathway and down-regulating the E6 and E7 expression in the tumor tissues in vivo.

Despite the promising anti-tumor effect of the co-expression vaccine on cervical cancer in mice model in this study, this delivery way still should be selected prudently in the future clinical trial. A previous phase I clinical trial showed that after injecting with high doses of the Salmonella typhimurium (VNP20009) through vein, the inability to fully colonize tumors in metastatic melanoma patients was different from that in rodent tumor models^37^. The reasons for the undesirable outcome might as follows: (a) Most of metastatic lesions of the patients were detected by fine-needle aspiration (FNA) whose sensitivity might have been limited by focal tumor colonization or bacterial concentration. (b) The hypoxia environment and necrosis of tumor might also influence the colonization of attenuated Salmonella typhimurium. However, the metastatic melanoma lesions of the patients in that study might lack of hypoxia environment and necrosis as compared with that in primary lesion. The effect of attenuated Salmonella typhimurium on primary solid tumor remains unclear. (c) The main pathway of infection of Salmonella typhimurium is mucosa^38^. Therefore, mucosal immune induced by PhoP/PhoQ was used in this study, which presented an effective immune response and delivery ability in mice. However, additional preclinical and clinical experiments are required to examine the safety and effectiveness of phoP/phoQ and its mucosal immune pathway.

In conclusion, a pcDNA3.1-HPV16-L1-siE6 plasmid carried by attenuated Salmonella phoP/phoQ was constructed for therapy of cervical cancer through a nasal mucosa vaccination way. As a DNA vaccine, it up-regulated the expression of HPV16-L1 in the xenograft, induced the production of HPV16-L1 antibody and immune cytokines in serum, and down-regulated the expression of E6/E7 gene in tumor tissues. The premise supported that phoP/phoQ-delivered co-expression plasmid is an attractive candidate for cervical cancer treatment and require an evaluation of its safety and immunogenicity in women volunteers.

## Materials and methods

### Cells and culture conditions

Human cervical cancer cells Siha and baby hamster kidney cells BHK were purchased from American Type Culture Collection (ATCC, USA). The cells were cultured in IMDM medium (Hyclone, USA) supplemented with 10% fetal bovine serum (FBS) (Gibco, USA) and maintained at 37 °C in 5% CO_2_ atmosphere.

### Mice

Six-week-old BalB/c female mice and BalB/c nu/nu female nude mice were purchased from the Institute of Laboratory Animals Science (China). All animals were housed at the Laboratory Animal Center of Jilin University, with freely access to food and water. The animal experiments were approved by the Institutional Animal Care and Use Committee of Jilin University (China). All animal care was in accordance with the recommendations in the ARRIVE guidelines and the Good Laboratory Practice Regulations.

### Plasmid construction and bacterial strains

To construct pcDNA3.1-HPV16-L1, genomic DNA solution of cervical cancer tissues was prepared as templates. HPV16-L1 open reading fame was amplified using primers P1, 5′-CGAAGCTTGCCACCATGGCTCTTTGGCTGCCTAGTG-3′, and P2, 5′-CCGGTACCTTACAGCTTACGTTTTTTGCG-3′. The production of PCR was cloned into HindIII and KpnI sites of pcDNA3.1(+) plasmid to construct pcDNA3.1-HPV16-L1. The new plasmid was transformed into attenuated Salmonella phoP/phoQ or Ty21a strains by electroporation.

The sequences of siRNA-E6A, siRNA-E6B and siRNA-E7 were shown in Table 1. The shRNA fragments were cloned into HindIII and BamHIsites of pGC-silencer vector to construct the pGC-siRNA plasmids including pGCsi-E6A, pCGsi-E6B and pCGsi-E7. The pGC-siScramble was served as a negative control.

**Table 1:** The sequences of siRNA-E6A, siRNA-E6B, siRNA-E7 and siRNA-Scramble.

	Sequence
siRNA-E6A (109–127)	5′-GATCC**TGTGTGTACTGCAAGCAACt**tcaagaga **GTTGCTTGCAGTACACACA**TTTTTTGGAAA-3′
siRNA-E6B (288–306)	5″-GATCC**CAGCAATACAACAAACCGT** ttcaagaga **ACGGTTTGTTGTATTGCTG**TTTTTTGGAAA-3′
siRNA-E7 (101–119)	5′-GATCC**AGGAGGATGAAATAGATGG** ttcaagaga **CCATCTATTTCATCCTCC**TTTTTTTGGAAA-3′
siRNA-Scramble	5′-GATCC**GTATAAGTCAACTGTTGAC** ttcaagaga **GTCAACAGTTGACTTATAC**TTTTTTGGAAA-3′

U6 promoter and siRNA-E6A fragment were amplified with primer P3, 5′-GCAGATCTTGCTTCGCGATGTACGGGCC-3′ and P4, 5′-GGTCGCGAGGGCTATGAACTAATGACCC-3′, and PCR production was cloned into BglII and NruI sites of pcDNA3.1-HPV16-L1 to generate pcDNA3.1-HPV16-L1-siE6 plasmid.

### Cell transfection

BHK cells were transfected with pcDNA3.1-HPV16-L1, and Siha cells were transfected with various pGC-siRNA plasmids using the Lipofectamine 2000 reagent (Invitrogen, USA).

### Immunocytochemical (ICC) staining

BHK cells were seeded to 24-well plates with cover slips on the bottom at 2 × 10^5^ cells/well and transfected with pcDNA3.1-HPV16-L1 for 72 h. Standard ICC procedures were carried out according to the protocol of manufactory (Beyotime, China). Rabbit anti-HPV16-L1 (1:50, RND, USA) was used as the primary antibodies and biotinylated goat anti-rabbit IgG was used as the secondary antibody.

### Immunofluorescence

Siha cells were seeded to 24-well plate at 2 × 10^5^ cells/well and transfected with pGC-siRNA plasmids for 48 h, standard immunofluorescence procedures were carried out as our previous study^39^. Rabbit anti-HPV16-E6/E7 (RND, USA) antibodies were used as the primary antibody. Goat anti-Rabbit IgG (H + L) conjugated with Alexa Fluor Plus 594 (Thermo Fisher) was used as the secondary antibody. The cover slips were analyzed using a fluorescence microscope (Olympus, Japan). Blue represented the nucleus stained with Hochest and red represented HPV16-E6 or HPV16-E7 protein.

### TUNEL assay

After transfection for 48 h, the cell apoptosis rates were assessed with terminal dUTP nick-end labeling (TUNEL) assay according to manufactory (Richoe, German)^40^. Sections were finally counter stained with hematoxylin.

### Flow cytometry

48 h after transfection with the pGC-siRNA plasmids, Siha cells were fixed with 70% cold ethanol at 4 °C for 12 h and stained with 1.5 mL (0.5 mg/L) propidium iodide (PI) solution. The flow cytometer (BD FACSCalibur, USA) was used for single parameter analysis. The apoptosis peak was presented as the hypodiploid peak in front of G1 peak. The cell cycles were calculated as well.

### MTT assay

MTT assay was performed as described previously^41^. Simply, Siha cells were seeded into 96-well plates at 2 × 10^3^ cells/well. After being transfected with the pGC-siRNA plasmids, MTT (Sigma, USA) solution (10 µL per well, 5 g/L) was added into each well. The absorbance was detected at 490 nm using a Microplate Reader (Japan).

### Western blot

Western blot was performed as described in previous study^17^. Rabbit anti-HPV16-L1, anti-HPV16-E6, anti-HPV16-E7 (R&D, USA), Rabbit anti-P53, anti-Bax, anti-Caspase9, anti-Caspase3, anti-cleaved-Caspase3 and anti-β-actin (Affinity, China) were used as the primary antibodies. After being incubated with secondary-HRP-antibodies (Promega, USA), the protein levels were detected with BCIP/NBT Alkaline Phosphatase Color Development Kit (Beyotime, China) and the densities of the specific bands were quantified with an imaging densitometer (Tanon, China).

### Semi-quantitative reverse transcription-PCR (RT-PCR)

Total RNA was extracted from the cells or tumor tissues using Trizol X-100 Reagent (Promega, USA). 5 μg of total RNA was used to reversely transcribed to cDNA using a commercially available RT-PCR kit (Promega, USA). The primers were shown in Table 2.

**Table 2:** The sequences of RT-PCR primers.

	Sequences
HPV16-E6	5′-CAGAGCTGCAAACAACTATACA-3′ 5′-CACCGACCCCTTATATTATG-3′
HPV16-E7	5′-GCATGGAGATACACCTACATTG-3′ 5′-TGGTTTCTGAGAACAGATGG-3′
p53	5′-CCTCCTCAGCATCTTATCCG-3′ 5′-ACAAAACACGCACCTCAAA-3′
bax	5′-AGGGTTTCATCCAGGATCGAGC-3′ 5′-AGGCGGTGAGGACTCCAGCC-3′
caspase9	5′-GAACTAACAGGCAAGCAGC-3′ 5′-GCATCCATCTGTGCCGTA-3′
caspase3	5′-AGAACTGGACTGTGGCATTG-3′ 5′-TTCTGTTGCCACCTTTCG-3′
GAPDH	5′-GAAGGTGAAGGTCGGAGTC-3′ 5′-GAAGATGGTGATGGGATTTC-3′

### Mice nasal mucosal immunity experiment

Female BalB/c mice were randomly divided into 5 groups (n = 6) and intranasally treated with the following dilution at the volume of 10 μL: (a) PBS, (b) phoP/phoQ (pcDNA3.1), (c) Ty21a (pcDNA3.1), (d) phoP/phoQ (pcDNA3.1-HPV16-L1), (e) Ty21a (pcDNA3.1-HPV16-L1). The treatment was performed on day 0, 7, and 21. Mice were performed euthanasia followed by AVMA guidelines on day 28. The serum and genital secretions were collected for ELISA assay.

Another group of Female BalB/c mice were randomly divided into 7 groups (n = 6) and intranasally treated with 10 μL phoP/phoQ (pcDNA3.1-HPV16-L1). The treatment was performed only on day 0. Mice were performed euthanasia followed by AVMA guidelines on day 0, 5, 10, 15, 20, 25, and 30.

### Xenografts tumor

Female BalB/c nu/nu nude mice were subcutaneously injected with 5 × 10^6^ Siha cells. When the tumors grown to a diameter of 3 mm, the mice were randomly split into 3 groups (n = 5), injected PBS, pGCsi-scramble and pGCsi-E6A (20 μg) respectively into tumor, and performed with electro-transfection twice. The treatment was repeated on day 15. The diameter of tumor was measured weekly. Mice were sacrificed on week 8 and the tumors were collected.

Other subcutaneous xenograft models were randomly split into 5 groups (n = 5), and intranasally administrated with the following dilution at the volume of 10 μL: (a) Mock (PBS), (b) Scramble (phoP/phoQ-pcDNA3.1), (c) siE6 (phoP/phoQ -pGCsi-E6A), (d) HPV16-L1 (phoP/phoQ -pcDNA3.1-HPV16-L1), (e) siE6 + L1 (phoP/phoQ -pcDNA3.1-HPV16-L1-siE6). The diameter of tumors was measured weekly. Mice were sacrificed on week 8 and the tumors and serum were collected.

### Histomorphological assay

Hematoxylin and eosin (H&E) stained was performed as described previously^41^. The images were subsequently acquired using a light microscope with 200× magnification.

### Immunohistochemical (IHC) staining

Immunohistochemistry was performed as described previously^17^. Histological slices from tumors were prepared for HPV16-L1, HPV16-E6 and HPV16-E7 IHC staining (Supplementary Information).

### ELISA assay

Anti-HPV16-L1 antibody was detected by an enzyme-linked immunosorbent assay (ELISA). The DuoSet ELISA Ancillary Reagent Kit (DY008, USA) was purchased from R&D company. The clear 96-well microplate was coated with 100 μL of purify HPV16-L1 protein at a concentration of 10 μg/mL. Seal the plate and incubate overnight at 4 °C. Then, the plate was washed with the wash buffer (0.05% Tween-20 in PBS, PBST). After blocking the plates with 1% BSA diluting in PBST, 50 μL of diluted serum (10 μL of each sample was diluted in 40 μL blocking buffer) was added to each well. The plates were incubated for 45 min at 37 °C. After washing four times, 50 μL of biotin-labeled goat anti-human IgG (V7830, Promega, China) was added for 30 min at 37 °C. After washing four times, 50 μL of the working dilution of streptavidin-HRP was added to each well for 30 min at 37 °C. After washing 4 times, each well was added with color reagent A and color reagent B. After 15 min of incubation in the dark at 37 °C, the reaction was stopped by the addition of 50 μL of stop solution to each well. The absorbance at 450 nm were detected with a Microplate Reader (BioTke, China). IL-2 and IFN-γ in serum and genital secretions were detected by ELISA kits according to the manufacturer’s instructions (M2000 for IL-2, MIF00 for IFN-γ, R&D, USA).

### Statistical analysis

Data was analyzed with the statistical software SPSS 17.0 and the results expressed as means ± SD. Statistical analysis were made for multiple comparisons using one-factor analysis of variance (ANOVA). P < 0.05 was considered as statistical differences.

## Supplementary Information

Supplementary Information.

## Data Availability

The datasets generated during and/or analysed during the current study are available from the corresponding author on reasonable request.
