# Supplementary material for: Attenuated Salmonella carrying plasmid co-expressing HPV16 L1 and siRNA-E6 for cervical cancer therapy
Source: Sci Rep. 2021 Oct 11;11:20083. doi: 10.1038/s41598-021-99425-3 (PMC8505555; doi:10.1038/s41598-021-99425-3)
Supplement: Supplementary file 1 — Supplementary Information. [file 41598_2021_99425_MOESM1_ESM.pdf]

Attenuated Salmonella carrying plasmid co-expressing HPV16 L1 and siRNA-E6 for cervical cancer therapy

Junyu Chen[1,2], Shuhua Zhao[2], Wenxi Tan[2], Taiwei Wang[1], Shan Wu[3], Changshuai Wang[1], Yu Jiang[1], Tuo Zhou[1], Zhuo Zhang[1], Lijing Zhao[1*]

[1]Department of Rehabilitation, School of nursing, Jilin University, Changchun, China

[2]Department of Gynecology, Second Hospital, Jilin University, Changchun, China

[3]Key Laboratory of Reproductive Genetics (Ministry of Education) and Department of Reproductive Endocri-nology, Women's Hospital, Zhejiang University, School of Medicine, Zhejiang, China

*Correspond to Lijing Zhao,

Email address: zhao_lj@jlu.edu.cn

Postal address: 965 Xinjiang Street, Changchun City, China.

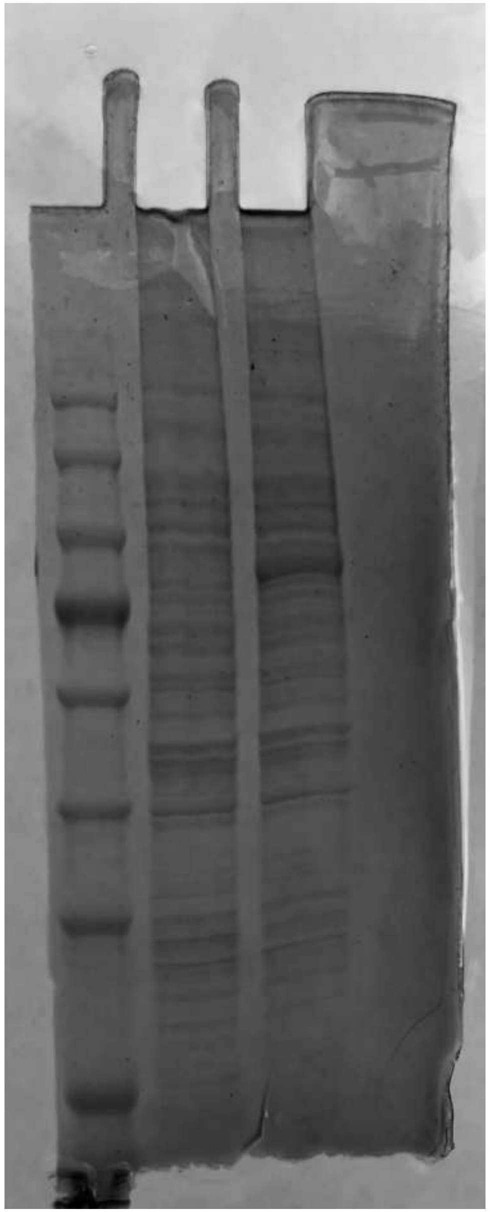

Figure 1C

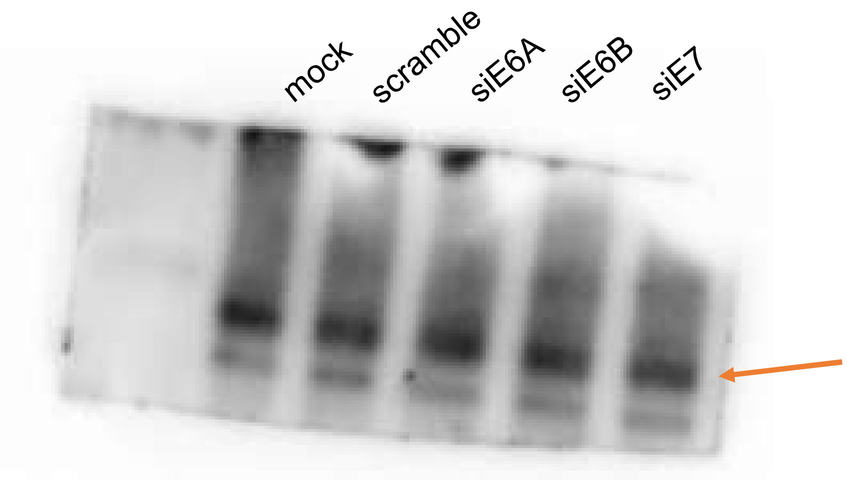

β-actin for the Cells figure 3C

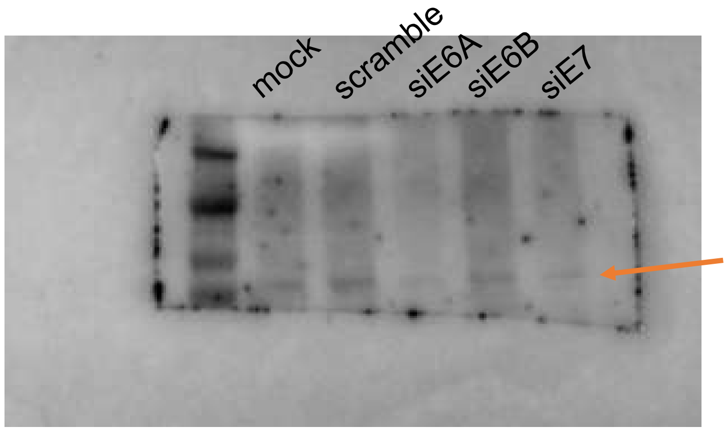

E6 for the Cells figure 3C

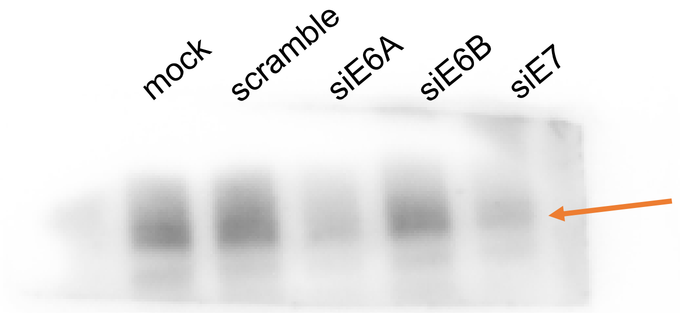

E7 for the Cells figure 3C

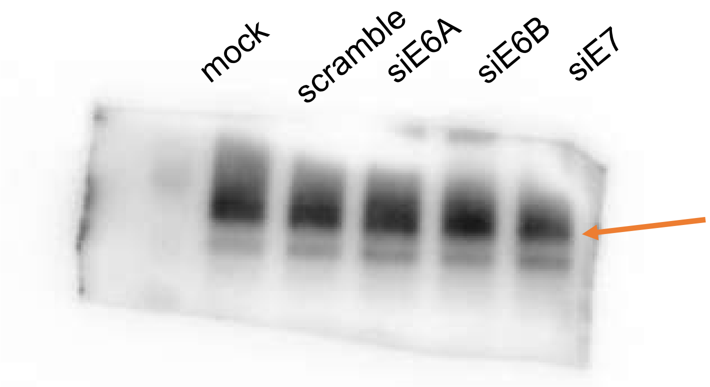

β-actin for the Cells figure 4G

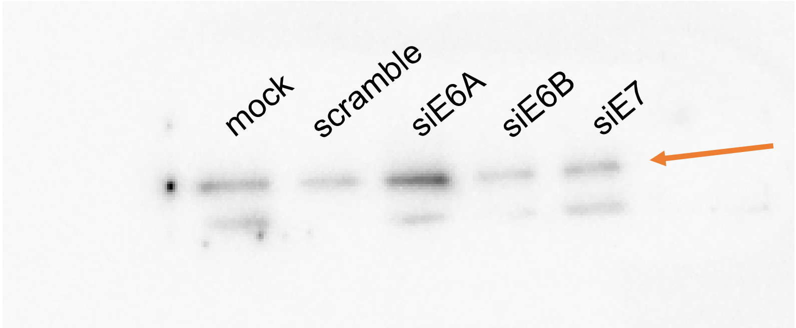

P53 for the Cells figure 4G

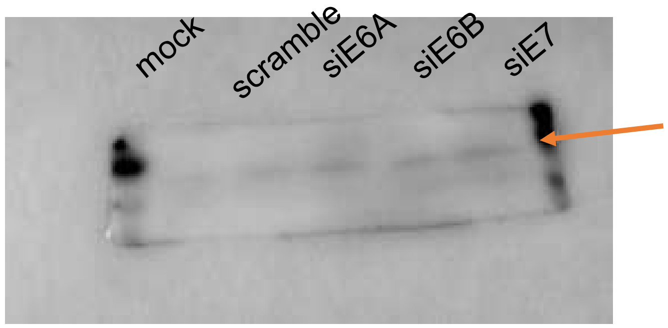

Bax for the Cells figure 4G

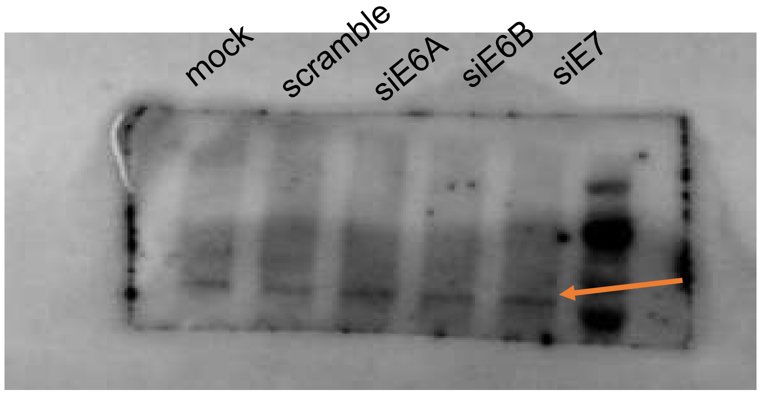

Caspase9 for the Cells figure 4G

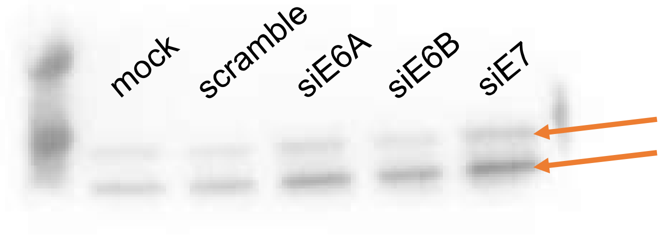

C-Caspase3 for the Cells figure 4G
